# Construction of microRNA and transcription factor regulatory network based on gene expression data in cardiomyopathy

**DOI:** 10.1186/s40001-014-0057-5

**Published:** 2014-10-24

**Authors:** Lei Wang, Jihua Hu, Haijian Xing, Min Sun, Juanli Wang, Qiang Jian, Hua Yang

**Affiliations:** Department of Cardiology, Xi’an Children’s Hospital, 69 Xi Ju Rd, Lianhu District, Xi’an, 710003 China

**Keywords:** Cardiomyopathy, Transcription factors, miRNAs, Gene expression

## Abstract

**Background:**

Cardiomyopathy is a progressive myocardial disorder. Here, we attempted to reveal the possible mechanism of cardiomyopathy at the transcription level with the roles of microRNAs (miRNAs) and transcription factors (TFs) taken into account.

**Method:**

We firstly identified differentially expressed genes (DEGs) between cardiomyopathy patients and controls with data from the gene expression omnibus (GEO) database. DEGs were associated with the canonical pathways, molecular and cellular functions, physiological system development and function in the Ingenuity Knowledge Base by using the Ingenuity Pathway Analysis (IPA) software. TFs and miRNAs that DEGs significantly enriched were identified and a double-factor regulatory network was constructed.

**Results:**

A total of 1,680 DEGs were identified. The DEGs were enriched for various pathways, with glucocorticoid receptor signaling as the most significant. A double-factor regulatory network was constructed, including seven TFs and two miRNAs. A subnetwork under the regulation of *MEF2C* and *SRF* was also constructed to illustrate their regulatory effects on cardiac functions.

**Conclusion:**

Our results may provide new understanding of cardiomyopathy and may facilitate further therapeutic studies.

## Background

Cardiomyopathy is a progressive myocardial disorder, usually leading to cardiovascular death or heart failure-related disability [1]. It develops at any age, in either sex, and in any population [2,3]. The etiology of cardiomyopathy is highly complex and improving its treatments has become a research hotspot.

Using bioinformatics combined with gene expression data to identify potential therapeutic targets has shown great application prospects. The majority of the previous studies mainly focused on the analyses of differentially expressed genes (DEGs), without considering microRNAs (miRNAs) and transcription factors (TFs) that regulate the expression of DEGs. miRNAs are small non-coding RNAs that control various biological processes through affecting the stability and translation of target mRNAs. Previous studies have proposed several miRNAs as being involved in the pathogenesis of cardiomyopathy, such as miR-1 [4] and miR-21 [5]. TFs can regulate gene expression through binding to the cis-elements in target genes’ promoter regions. TFs, such as *MEF2*, have been reported to be associated with cardiomyopathy [6]. Considering the important regulatory roles of miRNAs and TFs in the pathogenesis of cardiomyopathy, identification of miRNAs and TFs that enriched with target DEGs and construction of a double-factor regulatory network may provide new understanding of the molecular mechanism of cardiomyopathy.

In the current study, based on gene expression data from the Gene Expression Omnibus (GEO) database, we acquired DEGs, miRNAs and TFs that enriched with target DEGs and constructed a double-factor regulatory network. Our results may reveal the possible mechanism of cardiomyopathy at the transcription level.

## Methods

### Ethics Statement

This study was approved by the institutional review board of the Xi’an Children’s Hospital (20140518). Written informed consent was obtained from all patients for the publication of this report and any accompanying images.

### Microarray data

The gene expression profile GSE5406 from the GEO database was used. This dataset includes transcription profiles of 210 left ventricular myocardial tissue samples, 194 of which were from patients with advanced cardiomyopathy and 16 of which were from healthy donors. All patients had New York Heart Association class 3 to 4 symptoms and left ventricular systolic dysfunction, with ejection fraction of 14 ± 8% (mean ± SD). Patients suffered from heart failure due to ischemic (n =86) or idiopathic dilated (n =108) cardiomyopathy. Control samples had normal left ventricular function with ejection fraction of 56 ± 7% (*P* =0.0001 versus patients). None of the subjects received mechanical support with left ventricular assist devices. Myocardial tissue samples were obtained from patients undergoing heart transplantation and from controls deemed unsuitable for transplantation. Whole hearts were removed after preservation in cold cardioplegia at the time of transplantation or donor harvest. Then, segments of noninfarcted left ventricular free wall were snap-frozen in liquid nitrogen. For each sample, RNA was isolated by using Trizol reagent (Invitrogen, Carlsbad, CA, USA). The dataset was generated by using the (HG-U133A) Affymetrix Human Genome U133A Array (Affymetrix, Santa Clara, CA, USA).

### Identification of differentially expressed genes (DEGs)

Raw data from all arrays were normalized using Robust Multi-array Analysis (RMA) [7] in the R software (version 3.0.0). The resulting expression values were used to identify DEGs with the limma package (3.12.1) in R. DEGs were detected by using *t*-tests and multiple test corrections were carried out with the Benjamini-Hochberg method [8]. The threshold for significance was set as *P* <0.01.

### Enrichment analysis

To explore the functions and pathways of DEGs, DEGs were associated with the canonical pathways, molecular and cellular functions, physiological system development and function in the Ingenuity Knowledge Base by using the Ingenuity Pathway Analysis (IPA) software (Ingenuity® Systems, http://www.ingenuity.com).

### Construction of miRNA-TF regulatory network

We also acquired miRNAs and TFs which were overrepresented with target DEGs. For miRNA analysis, we used Targetscan [9], miRanda [10] and Pita [11] for miRNA target DEGs prediction. To avoid false positive results, miRNA-DEG prediction results supported by all three prediction methods were considered to be confidential. For TF analysis, we obtained TF binding sites and the coordinate’s position information of human being from the University of California Santa Cruz (UCSC) database. TFs and miRNAs that DEGs significantly enriched were identified by using the hypergeometric distribution test. *P* <0.01 was set as the threshold. Based on the regulatory relationship between miRNAs or TFs and DEGs, a network was constructed by using the IPA software.

## Results

A total of 1,680 DEGs were identified, including 963 down-regulated genes and 717 up-regulated ones in advanced cardiomyopathy patients.

**Figure 1:**
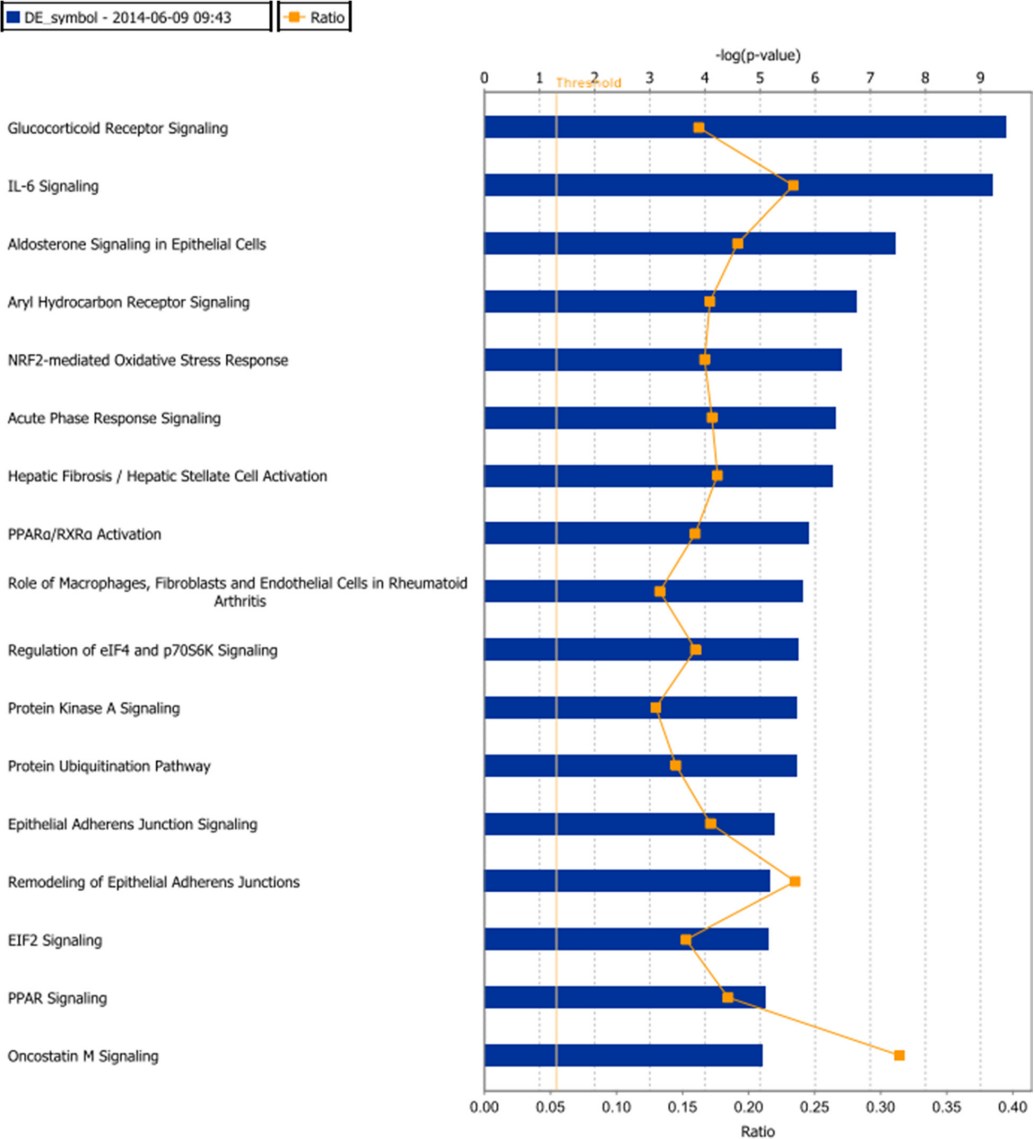
**Ingenuity Pathway Analysis results.** The significant canonical pathways in which differently expressed genes (DEGs) were enriched are shown.

As shown in Figure 1, the IPA results of canonical pathways showed that the DEGs were enriched for various pathways, among which glucocorticoid receptor signaling was the most significant. Molecular and cellular functions analysis revealed that ‘Cell Death and Survival’ (*P* =2.53 × 10^−24^ to 1.36 × 10^−3^) was the top molecular function affected by DEGs followed by ‘Cellular Growth and Proliferation’ (*P* =3.37 × 10^−24^ to 1.36 × 10^−3^) (Table 1). ‘Cardiovascular System Development and Function’ (*P* =4.17 × 10^−12^ to 1.20 × 10^−3^) was the top physiological function mediated by DEGs (Table 1).

**Table 1:** **Ingenuity Pathway Analysis: functions related to differentially expressed genes**

**Name**	***P*** **-value**	**Number of molecules**
Molecular and Cellular Functions		
Cell Death and Survival	2.53 × 10^−24^ to 1.36 × 10^−3^	405
Cellular Growth and Proliferation	3.37 × 10^−24^ to 1.36 × 10^−3^	414
Cellular Movement	5.41 × 10^−13^ to 1.36 × 10^−3^	236
Gene Expression	8.14 × 10^−11^ to 1.20 × 10^−3^	247
Cellular Development	1.13 × 10^−10^ to 1.36 × 10^−3^	328
Physiological System Development and Function		
Cardiovascular System Development and Function	4.17 × 10^−12^ to 1.20 × 10^−3^	125
Organismal Development	3.96 × 10^−11^ to 1.22 × 10^−3^	92
Tissue Development	3.68 × 10^−8^ to 1.22 × 10^−3^	163
Tumor Morphology	3.84 × 10^−7^ to 1.20 × 10^−3^	117
Connective Tissue Development and Function	8.52 × 10^−6^ to 1.24 × 10^−3^	40

**Figure 2:**
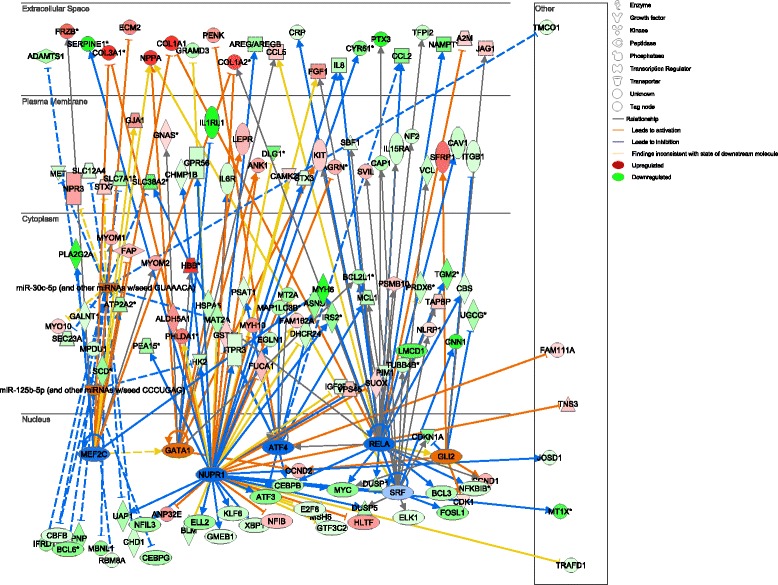
**The microRNA (miRNA) and transcription factor (TF) regulatory network.** TFs are shown with ellipse and miRNAs are shown with semicircle. Up-regulated genes are shown in red and down-regulated genes are shown in green.

**Figure 3:**
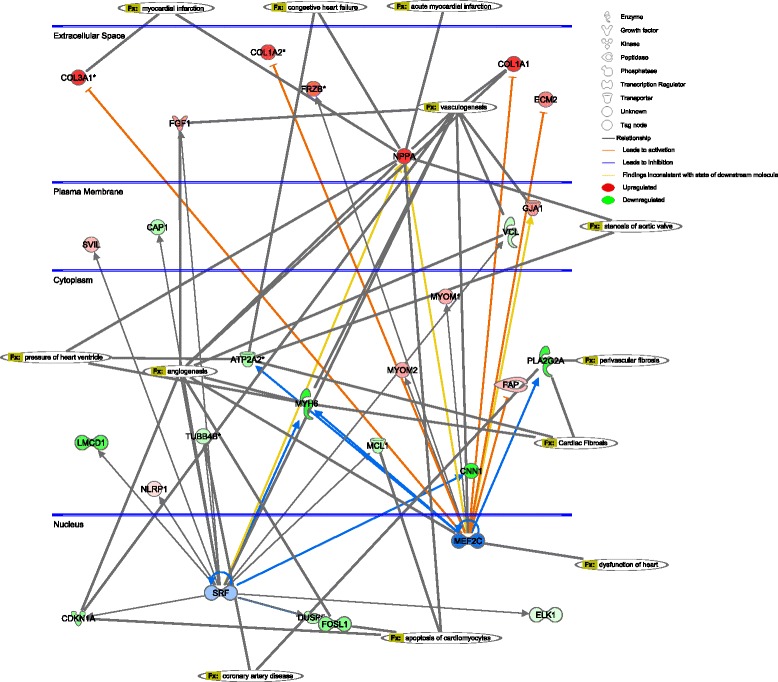
**The network under the regulation of** ***SRF*** **and** ***MEF2C*** **.** Up-regulated genes are shown in red and down-regulated genes are shown in green. The related cardiac functions are indicated in long ellipses.

The results of miRNA and TF enrichment analysis are listed in Table 2. A network was constructed to illustrate the regulatory relationships (Figure 2). Considering the important role of *SRF* and *MEF2C* in the progression of cardiomyopathy, a subnetwork under their regulation was constructed and the biological functions they may affect were also indicated (Figure 3).

**Table 2:** **Transcription factors and microRNAs (miRNAs) enriched with target differentially expressed genes (DEGs)**

**Name**	***P*** **-value**
MicroRNA	
miR-30c-5p (and other miRNAs w/seed GUAAACA)	3.12 × 10^−4^
miR-125b-5p (and other miRNAs w/seed CCCUGAG)	2.15 × 10^−3^
Transcription factors	
MEF2C	5.65 × 10^−6^
GATA1	6.11 × 10^−5^
SRF	1.53 × 10^−4^
NUPR1	4.18 × 10^−4^
ATF4	7.25 × 10^−4^
RELA	9.35 × 10^−3^
GLI2	1.42 × 10^−2^

## Discussion

In the current study, based on GSE5406 from the GEO database, 1,680 DEGs were identified between advanced cardiomyopathy patients and healthy controls. DEGs were mapped into the Ingenuity Knowledge Base and several canonical pathways were screened out, with glucocorticoid receptor signaling as the most significant. Glucocorticoid receptors are involved in cardiovascular homeostasis [12] and glucocorticoid could protect rodent hearts from ischemia/reperfusion injury [13]. Our results confirmed the essential roles of this pathway in the pathogenesis of cardiomyopathy. Molecular and cellular functions analysis revealed that ‘Cell Death and Survival’ (*P* =2.53 × 10^−24^ to 1.36 × 10^−3^) was the top molecular function affected by DEGs, suggesting that dysregulation of the cell function may contribute to the progressive heart failure induced by cardiomyopathy.

TFs or miRNAs that DEGs significantly enriched were identified and a double-factor regulatory network was constructed, including seven TFs and two miRNAs (Figure 2). All these miRNAs and TFs have previously been implicated in cardiomyogenesis or cardiac function. Considering their important regulatory roles in controlling various biological processes, these miRNAs and TFs may provide new avenues for the therapeutic strategies of cardiomyopathy. In addition, since all these miRNAs and TFs were detected in patients suffered from New York Heart Association class 3 to 4 symptoms, they may also be considered as prognosis markers in clinical practice. A subnetwork under the regulation of *SRF* and *MEF2C* was also constructed and the functions they may affect were also indicated (Figure 3) considering the considerable evidence in support of their involvement in cardiomyopathy [14-17]. As shown in Figure 3, both *SRF* and *MEF2C* may inhibit the expression of *MYH6* and *CNN1. MYH6* is a famous cardiac muscle myosin gene and mutations of this gene are implicated in cardiomyopathy in various studies [18]. Inhibition of *CNN1* expression may disrupt its suppression of cardiomyopathy through the εPKC pathway [19]. In addition, both *SRF* and *MEF2C* may regulate the expression of *NPPA*, which was overexpressed in patients in our results. This gene is involved in many cardiac dysfunctions (Figure 3), including myocardial infarction, heart failure, and high ventricular pressure [20,21].

## Conclusion

In summary, with DEGs identified by gene chip data generated by high-throughput technologies from the GEO database, we constructed a cardiomyopathy double-factor regulatory network and revealed the possible mechanism on regulation level. A subnetwork under the regulation of *SRF* and *MEF2C* was also constructed to illustrate their possible regulatory roles. Our results may facilitate further therapeutic studies of cardiomyopathy.

## Abbreviations

DEG: differentially expressed genes
GEO: Gene Expression Omnibus
IPA: Ingenuity Pathway Analysis
miRNAs: microRNAs
RMA: Robust Multi-array Analysis
TF: transcription factor
UCSC: University of California Santa Cruz
